# Hematological and Biochemical Parameters at Admission as Predictors for Mortality in Patients with Moderate to Severe COVID-19

**DOI:** 10.4314/ejhs.v33i2.3

**Published:** 2023-03

**Authors:** Danis Pertiwi, Maritsatun Nisa, Andina Putri Aulia, Rahayu

**Affiliations:** 1 Department of Clinical Pathology of Faculty of Medicine, Universitas Islam Sultan Agung; 2 Faculty of Medicine, Universitas Islam Sultan Agung; 3 Department of Clinical Microbiology of Faculty of Medicine, Universitas Islam Sultan Agung

**Keywords:** NLR, SARS-CoV-2, c-reactive protein, D-dimer

## Abstract

**Background:**

Timely diagnosis and effective use of available resources are urgent to avoid the loss of time, medical, and technological resources, particularly in COVID-19 pandemic. This study aimed to identify the most dominant predicting factor for mortality in moderate-severe COVID-19 patients.

**Methods:**

This retrospective cohort study included a total of 253 patients diagnosed with moderate-severe COVID-19. The primary outcome measure was mortality during hospitalization. The receiver operating characteristic (ROC) curve was used to determine cut-off points. The data were categorized according to the cut-off points in ROC curve and analyzed using Chi-square and by binary logistic regression test to identify the independent predictors associated with mortality.

**Results:**

The mean number of leukocytes (/µL), neutrophils (%), neutrophil lymphocyte ratio (NLR), platelet lymphocyte ratio (PLR), C-reactive protein (CRP, mg/L), and D-dimer (mg/L) in the non-survived group was significantly higher than those of the survived group. Meanwhile, the mean number of platelet count/µL, absolute lymphocyte count (ALC), in the non-survived group was significantly lower than those of the survived group. CRP level predicted mortality with a cut-off point of ≥8.41 mg/L, sensitivity of 98.1%, and specificity of 72.0% (P = .000).

**Conclusions:**

High leukocyte count, low platelet count, high NLR, high CRP level, and high D-dimer on admission predicted mortality of COVID-19 patients. In addition, CRP was found to be the most dominant predicting factor of mortality in moderate-severe COVID-19 patients.

## Introduction

Coronavirus disease 2019 (COVID-19) caused by SARS-CoV-2 is an infectious disease that affects the respiratory system and was declared by WHO a pandemic in 2020. The number of confirmed cases and deaths is increasing worldwide with detrimental impacts on various fields. Two months after hitting India, the delta variant hit Indonesia and increased confirmed cases as well as deaths of COVID-19(1). The Ministry of Health of Republic Indonesia has made efforts to improve the response to the pandemic by referring to the guidelines from the World Health Organization (WHO) (2).

SARS-CoV-2 invasions depend on the receptor-binding between the Angiotensin Converting Enzyme 2 (ACE2) and the SARS-CoV-2 S protein. ACE2 is located in various organs, such as the epithelial cells of the lungs, heart, renal system, and gastrointestinal tract. After an incubation process that lasts 3–14 days, the virus spreads through the bloodstream which eventually causes clinical manifestations, especially in the lungs and gastrointestinal tract (3). In some cases, a “cytokine storm” occurs, which is an increase in proinflammatory cytokines that can cause microvascular changes. This condition leads to disseminated intravascular coagulation (DIC) and pulmonary embolism which can worsen the clinical condition and death (4).

Covid-19 infection usually starts with flu like symptoms (5) and can be asymptomatic or may have a mild to severe course (6). Association between hemogram parameters and Covid-19 infection has been studied and NLR (7) was found to be related with the infection. Moreover, red cell distribution width, marker of anisocytosis in hemogram, has been associated with recurrent hospitalizations in patients with Covid-19 (8). Other inflammatory markers were introduced as predictors of frailty in diabetics during Covid-19(9). Therefore, it can be assumed that inflammatory indices could be associated with Covid-19 infection. NLR is also associated with other inflammatory conditions such as thyroid conditions (10), irritable bowel disease (11), thyroiditis (12), and Covid-19 infection (7). Various changes in simple hematological and biochemical parameters can indicate the aggravation of the disease. A previous study proved that some hematological and biochemical parameters can be used as predictors of mortality in COVID-19 patients (13). The recovery and mortality rate is often associated with abnormalities such as leukocyte, platelet, Neutrophil, and Absolute Lymphocyte Count (ALC), as well as Neutrophil Lymphocyte Ratio (NLR), Platelet Lymphocyte Ratio (PLR), C-reactive Protein (CRP) levels, and D-dimer (14–16). Other studies also proved that NLR and PLR are predictors of severity (13), while Yang et al. mentioned that among the various hematological parameters, only NLR can be used as predictor of mortality (17).

This study aimed to identify hematological parameters such as Leukocyte, Platelets, and Neutrophil count, as well as ALC, PLR, and NLR, along with biochemical parameters namely CRP and D-dimer as predictors for mortality in moderate-severe COVID-19 patients. The findings can be used as a basis for managing COVID-19 patients more comprehensively and efficiently.

## Subjects and Methods

**Design**: This retrospective cohort study received permission from the ethics committee of Sultan Agung Islamic Teaching Hospital (No.208 / KEPK / 2021); a referral hospital for COVID-19 patients.

**Study population**: The data were obtained from medical records of patients confirmed with COVID-19 using the consecutive sampling technique from October 2020 to June 2021. The inclusion criteria were adult patients confirmed positive for COVID-19 based on rt-PCR results, moderate to severe/critical hospitalized patients, and aged ≥18 years. Meanwhile, the exclusion criteria were pregnant women, post-operative patients, patients with malignancy, autoimmune diseases, immunodeficiency diseases, burns, hematological disorders, and those referred from other hospitals and had received previous therapy. The evaluated variables were the number of leukocytes, platelets, neutrophils, ALC, NLR, PLR, CRP, and D-dimer which were examined prior to hospital admission. All patients received therapy in accordance to the signs and symptoms defined by the COVID-19 Management Guidelines 4th Edition in 2022 (18). The primary outcome of this study was mortality during hospitalization.

**Statistical analysis**: After the data were collected, the normality test was carried out with a numeric scale using the Kolmogorov Smirnov test. Data that were not normally distributed were tested using the Mann-Whitney test, while those with normal distribution were tested using the t-independent test (p< 0.05 is significant). ROC curve was carried out to determine the cut-off points for predicting mortality. Furthermore, the data were categorized according to the cut-off points in ROC curve and then statistically evaluated using Chi-square and binary logistic regression tests. All statistical analyses were performed using IBM SPSS 25.

## Results

A total of 344 patients confirmed with COVID-19, however 91 patients were excluded (52 were pregnant, 23 were post operative patients, and 16 patients were referred from other hospitals and had received previous therapy) and only 253 patients met the study inclusion criteria. Patients were categorized into 2 outcome groups, those were survived (200 patients) and non-survived (53 patients). Background characteristics of participants is presented in Table 1. There was a significant difference of comorbid and types of comorbid between the groups.

**Table 1:** Demographic characteristics of COVID-19 patients in the study

Characteristics	Survived (n = 200 (79%))	Non-survived (n = 53 (21%))	*P* value
**Age**			
18–59	155 (81.2%)	36 (18.8%)	*P* = .150 a
≥ 60	45 (72.6%)	17 (27.4%)	
**Gender**			*P* = .068^a^
Women (n=114; 45%)	96 (84.2%)	18 (15.8%)	
Men (n=139; 55%)	104 (74.8%)	35 (25.2%)	
**Cough**			*P* = .519 a
Yes	173 (79.7%)	44 (20.3%)	
No	27 (75.0%)	9 (25%)	
**Diarrhea**			*P* = .958 a
Yes	4 (80%)	1 (20%)	
No	196 (79.0%)	52 (21.0%)	
**Shortness of breath**			*P* = .389 a
Yes	151 (77.8%)	43 (22.2%)	
No	49 (83.1%)	10 (16.9%)	
**Nausea and/Vomiting**			*P* = .299 a
Yes	33 (73.3%)	12 (26.7%)	
No	167 (80.3%)	41 (19.7%)	
**Anosmia**			*P* = .139 a
Yes	8 (100%)	-	
No	192 (78.4%)	53 (21.6%)	
**Painful swallowing**			*P* = .841 a
Yes	3 (1.5%)	1 (1.9%)	
No	197 (98.5%)	52 (98.1%)	

**Comorbid**			*P* = .019 a
Yes	124 (62%)	42 (79.2%)	
Hypertension (HT)	36 (87.8%)	5 (12.2%)	*P* = .002 a
Diabetes Mellitus (DM)	22 (68.8%)	10 (31.3%)	
HT dan DM	46 (78.0%)	13 (22.0%)	
Asthma	3 (100%)	-	
Others (Kidney, Heart)	17 (54.8%)	14 (45.2%)	
No	76 (38%)	11 (20.8%)	
**Radiology results**			*P* = .290 a
Bronchopneumonia	197 (79.4%)	51 (20.6%)	
Non-Bronchopneumonia	3 (60%)	2 (40%)	

a Chi-square

The hematological and biochemical examination results were presented in Table 2. The mean of leukocyte counts/µL, neutrophils (%), NLR, PLR, CRP (mg/L), and D-dimer (mg/L) in the non-survived group were significantly higher than those of survived group. Meanwhile, the platelet counts/µL, and ALC in the non-survived group were significantly lower than those of survived group.

**Table 2:** Hematological and biochemical parameters in COVID-19 patients

Variable	Survived	Non-survived	*P* value
Leukocytes count (/µL)	8814.7 ± 4572.6	12304.3 ± 6333.8	*P* = .000 b
Platelet counts (x10^3^/µL)	354.13 ± 121.2	271.17 ± 119.5	*P* = .000 ^c^
NLR	4.6 ± 9.27	11.7 ± 7.84	*P* = .000 b
ALC (/µL)	1941.2 ± 1202.7	1072.5 ± 579.7	*P* = .000 b
D-dimer (mg/L)	1.18 ± 2.0	6.63 ± 10.6	*P* = .000 b
CRP (mg/L)	13.04 ± 31.52	95.42 ± 82.86	*P* = .000 b
PLR	224.72 ± 154.90	321.62 ± 209.98	*P* = .001 b
Neutrophil (%)	67.05 ± 45.23	81.30 ± 9.06	*P* = .000 b

b Mann Whitney test

t independent test, NLR= Neutrophils Lymphocyte Ratio, ALC= Absolute Lymphocyte Count, CRP= C-Reactive Protein, PLR= Platelets Lymphocyte Ratio

**Figure 1:**
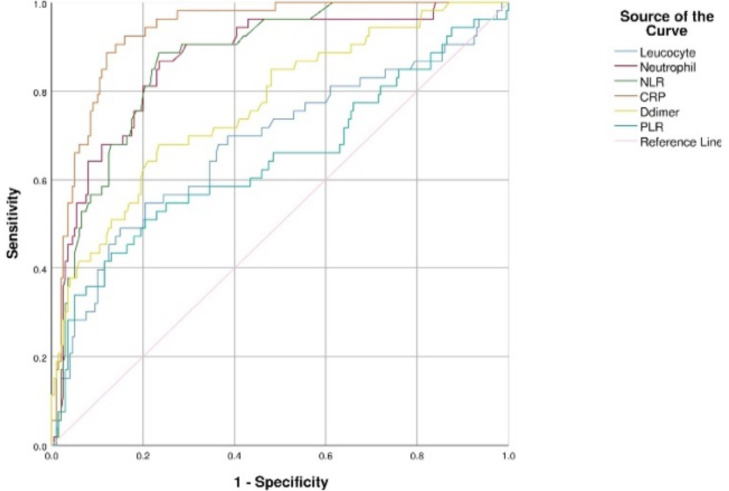
ROC curve for leukocytes, and neutrophils Count, NLR, PLR, CRP, and D-Dimer.

**Figure 2:**
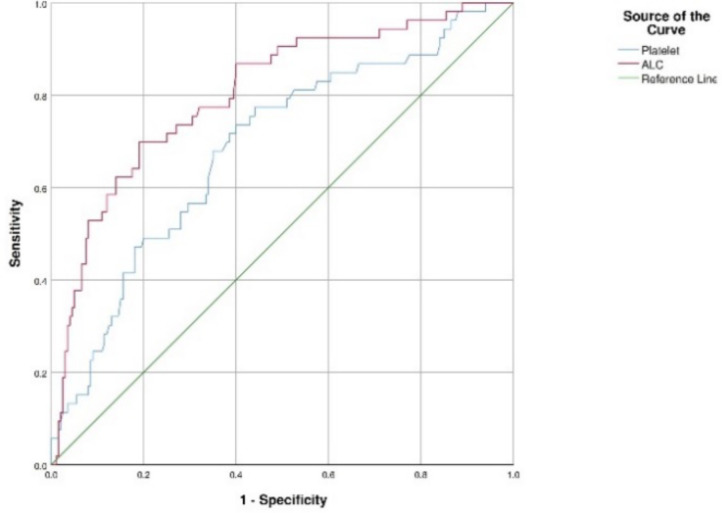
ROC curve for platelets counts and ALC.

The ROC Curve analysis results used to determine cut-off points on hematological and biochemical parameters were presented in figures 1 and 2. In the ROC curve analysis, it was found that hematological and biochemical parameters AUC were above 50%, therefore those can be used as predictors of mortality (Table 3). Based on the analysis, the best predictor of mortality was CRP with cut-off point, sensitivity, specificity, and AUC of ≥8.41, 98.1%, 72.0%, and 0.933 respectively.

**Table 3:** Cut-off points for hematological and biochemical parameters

Marker	Cut-Off Point	Sensitivity	Specificity	AUC	*P-value*	*95% CI*
Platelets (x10^3^/µL) *	315.5	71.7%	61.5%	0.687	.000	0.606 – 0.768
Leukocytes (/µL) **	11,075	54.7%	79.5%	0.682	.000	0.592 – 0.772
Neutrophils (%) **	66.05	96.2%	57.0%	0.870	.000	0.815 – 0.924
ALC (/µL) *	1,597	86.8%	60.0%	0.804	.000	0.737 – 0.872
NLR**	3.85	90.6%	71.5%	0.872	.000	0.824 – 0.920
PLR**	202.62	66. 0%	51.5%	0.645	.001	0.550 – 0.739
CRP (mg/L) **	8.41	98.1 %	72.0%	0.933	.000	0.901 – 0.965
D-dimer (mg/L) **	0. 67	81. 1%	53.5%	0.769	.000	0.695 – 0.843

* positive when the value is less than or equal to

** positive when the value is greater than or equal to

NLR= Neutrophils Lymphocyte Ratio, ALC= Absolute Lymphocyte Count, CRP= C-Reactive Protein, PLR= Platelets Lymphocyte Ratio

The bivariate statistical analyses (Tables 4) showed that there was a correlation between mortality and hematological-biochemical parameters in COVID-19 patients. Multivariate statistical analyses (Table 5) indicated that CRP was the most dominant predictor of mortality (RR =138.269; 95% CI =16.021 — 1193.296, *P* = .000).

**Table 4:** Bivariate analysis for hematological and biochemical parameters based on cut-off points

Variable	Survived (n = 200 (79%))	Non-survived (n = 53 (21%))	*P* value
**Leukocytes (/µL)**			
≥11,075	41 (58.6%)	29 (41.4%)	*P* = .000 a
<11,075	159 (86.9%)	24 (13.1%)	
**Platelets (x10^3^/µL)**			
<315.5	77 (67.0%)	38 (33.0%)	*P* = .000 a
≥315.5	123 (89.1%)	15 (10.9%)	
**Neutrophils (%)**			
≥66.05	86 (62.8%)	51 (37.2%)	*P* = .000 a
<66.05	114 (98.3%)	2 (1.7%)	
**NLR**			
≥3.85	57 (54.3%)	48 (45.7%)	*P* = .000 a
<3.85	143 (96.6%)	5 (3.4%)	
**ALC (/µL)**			
<1597	80 (63.5%)	46 (36.5%)	*P* = .000 a
≥1597	120 (94.5%)	7 (5.5%)	
**PLR**			
≥202.62	97 (73.5%)	35 (26.5%)	*P* = .023 a
<202.62	103 (85.1%)	18 (14.9%)	
**D-dimer (mg/L)**			
≥0.67	95 (68.8%)	43 (31.2%)	*P* = .000 a
<0.67	105 (91.3%)	10 (8.7%)	
**CRP (mg/L)**			
≥8.41	56 (51.9%)	52 (48.1%)	*P* = .000 a
<8.41	144 (99.3%)	1 (0.7%)	

a Chi-square test

NLR= Neutrophils Lymphocyte Ratio, ALC= Absolute Lymphocyte Count, CRP= C-Reactive Protein, PLR= Platelets Lymphocyte Ratio

**Table 5:** Multivariate analysis of hematological and biochemical parameters

Variable	Exp B	95% CI	*P* value
Leukocytes (/µL)	4.500	1.411 – 14.353	*P* = .011 a
Platelets (x10^3^/µL)	5.686	1.919 – 16.847	*P* = .002 a
NLR	8.262	2.492 – 27.397	*P* = .001 a
D-dimer (mg/L)	2.754	0.921 – 8.238	*P* = .070 a
CRP (mg/L)	138.269	16.021 – 1193.296	*P* = .000 a

a binary logistic regression test

NLR= Neutrophils Lymphocyte Ratio, CRP= C-Reactive Protein

## Discussion

After being declared as a global pandemic by the WHO, COVID-19 has infected millions of people and caused several deaths as well as chaos in the national health systems of various countries (1). The scarcity and limitations of medical equipment and resources have been experienced worldwide, even in developed countries, due to the enormous pressures on health systems caused by the rapid COVID-19 spread and the associated burden of the illness (19,20). In developing countries such as Indonesia, COVID-19 is a huge burden for the national health system due to the lack of medical facilities and resources to diagnose and treat patients. Therefore, it is very important to diagnose the disease in a timely and effective manner with the available resources to avoid the waste of time, medical, and technological resources (2).

Various combinations of hematological parameters have been used to predict the prognosis of COVID-19 cases. The multivariate statistical analysis reported that Leukocytes, Platelets count, NLR, D-dimer, and CRP predicted mortality of COVID-19 patients, and CRP was the best predictor for mortality.

In this study, the number of leukocytes was higher in the non-survived group and appeared to significantly affect mortality. This is in line with a study conducted by Khalid et al. where leukocytosis was found in the non-survived group (13). Açıksarı et al. also found that the incidence of leukocytosis was more frequent in the non-survived group (21). Based on the results, the Leukocyte count's cut-off point ≥11,075/µL (AUC = 0.682, sensitivity 54.7%, specificity 79.5%, *P* = .000) can be used as a predictor of mortality in COVID-19 in this study. Our finding was in contrast with Bastug's et al study which reported that leukocytes can be used as a predictor of mortality in COVID-19 with cut-off point ≥6,005 /µL (AUC = 0.769, sensitivity 77.8%, specificity 60.7%, *P*< .001) (14).

In several previous studies, patients with severe cases had leukocytosis, but there was a decrease in the number of lymphocytes (22). Meanwhile, lymphocytes play an important role in the regulation of cellular immunity. In COVID-19 patients, lymphopenia is often found, possibly due to the incidence of a cytokine storm. This refers to an increase in pro-inflammatory cytokines such as TNFα and IL-6 which might have a role in lymphopenia caused by T cell apoptosis (23). Diao *et al.*, proved that there was a correlation between the number of lymphocytes and the levels of TNFα and IL-6 in serum. Changes in the number of lymphocytes cause dysregulation of the immune system by inducing cytokine and chemokine responses which can also cause a cytokine storm culminating in multiple organ dysfunctions (24). The inability of the adaptive immune system to carry out virus eradication causes immune hyperactivity with an increase in inflammatory mediators, lymphopenia, or lymphocytic dysfunction occurring as compensation which eventually leads to a cytokine storm. Moreover, cytokine storm effects on organ systems cause damage that can lead to death (25). In this study, ALC or absolute lymphocyte count was lower in the deceased group, and had a significant effect on mortality. The cut-off point for ALC was determined ≤1,597/µL (AUC = 0.804, sensitivity 86.8%, specificity 60.0%, p = 0.000) as a predictor of mortality in COVID-19 in this study. Our finding was consistent with previous study by Barrett, et al. which reported that lower ALC correlated with mortality in COVID-19 cases, (26) while Tardón recorded a significantly low lymphocyte count in the non-recovered group (27).

Neutrophils and lymphocytes play an important role in innate and cellular/inflammatory immune responses. The high NLR reflects an imbalance between both responses, and this can be used as a severity indicator (28). In a meta-analysis of 15 studies, neutrophilia, lymphopenia, and elevated NLR were found in severe cases of COVID-19 (29). The NLR in this study was higher in the non-survived group, and had a significant effect on mortality. This is in line with the study of Assal, et.al where a significantly higher NLR was found in the non-survivor group (30). Furthermore, the NLR cut-off point was decided ≥3.85 (AUC = 0.872, sensitivity 90.6%, specificity 71.5%, *P* = .000) as a predictor of mortality in COVID-19 in this study. This finding was in contrast with previous study by Khalid et al which reported the cut-off point for NLR was ≥2.98 (AUC = 0.837; sensitivity 75%, specificity 61%, *P*<.001). The cut-off points as well as sensitivity and specificity were lower than those of in our findings (13).

Thrombocytopenia is a marker of the COVID-19 severity; this is because platelets have an important role in the inflammatory response associated with endothelial damage. The interaction between leukocytes and the proinflammatory cytokine activity of platelets leads to the release of cytokines (31). Based on the results, the platelet count was lower in the non-survived group, and had a significant effect on mortality. The Platelet cut-off point ≤315,500/µL (AUC = 0.687, sensitivity 71.7%, specificity 61.5%, *P* = .000) can be used as a predictor of death in COVID-19 in this study. Our finding was consistent with Mousavi et al. study which stated significantly lower platelet count in the deceased group (32).

D-dimers are small protein fragments that are released into the blood when a blood clot is degraded through fibrinolysis. Cases including deep vein thrombosis (DVT), pulmonary embolism, arterial thrombosis, disseminated intravascular coagulation, and conditions such as pregnancy, inflammation, cancer, chronic liver disease, post-traumatic, surgical status, and vasculitis can lead to an increase in D-dimer plasma levels (33). In this study, the D-dimer was significantly higher in the non-survived group than in the survived, and a correlation was found with mortality. Zhou, et al. reported that the high mortality in COVID-19 patients was due to high levels of D-dimer (34), while Tang, et al. reported that those with severe conditions had D-dimer values 3.5 times higher than other patients (35). The D-dimer cut-off point ≥0.67mg/L (AUC = 0.769, sensitivity 81.1%, specificity 53.5%, *P* = .000) was used as a predictor of mortality in COVID-19 in this study. This finding was in contrast with Yao's et al. study which informed an even higher cut-off point for D-dimer >2.14 mg/L (AUC = 0.85, sensitivity 88.2%, specificity 71.3%, *P* = .000) (36).

The findings revealed that CRP was the most dominant predictor of mortality within COVID-19 cases. CRP was significantly higher in the non-survived group, and a correlation was found with mortality. The role of CRP in disease pathology might involve host defense and inflammation. In response to the onset of inflammation (such as diabetic nephropathy (37), thyroiditis (38), and hepatitis (39)), CRP binds to pathogens and promotes their elimination by phagocytic cells, serving as the first line of innate host defense. In addition, CRP might exhibit anti-inflammatory effects by inhibiting neutrophil chemotaxis or exert a proinflammatory effect by increasing the expression of adhesion molecules as well as IL-1, IL-6, IL-8, and TNF-. Moreover, a recent work reported association between Covid-19 mortality and CRP based inflammatory markers (40). Elevated CRP serum levels in COVID-19 patients might indicate excessive inflammatory stress contributing to severe/critical illness or even death (41). The cut-off point of CRP ≥ 8.41 mg/dL (AUC = 0.933, sensitivity 98.1%, specificity 72.0%, *P* = .000) can be used as a predictor of death in COVID-19 in this study. A previous study by Luo et al found a much higher cut-off point of CRP, that was ≥ 41.4 mg/dL (AUC = 0.896, sensitivity 90.5%, specificity 77.6%, *P* < .001). It was also reported that CRP was a predictor for severe/critical illness in COVID-19 (41).

In conclusion, hematological and biochemical parameters can be used as predictors for mortality in COVID-19 patients. Their leukocyte, platelet, and neutrophil count, as well as ALC, NLR, PLR, CRP, and D-dimer reported at the admission might predict their mortality. In addition, the best predictor of mortality in COVID-19 patients is CRP. Despite using a cohort retrospective design with over 250 patients as subjects and having homogenic characteristics between the subject, this study was single centered. Further studies can be improved using more than just one center and using the parameters to predict the risk of ICU transferred, instead of mortality predictors.
